# Polystyrene Microplastics‐Induced Thyroid Dysfunction in Mice: A Study of Gene Expression, Oxidative Stress, and Histopathological Changes

**DOI:** 10.1002/vms3.70393

**Published:** 2025-05-14

**Authors:** Md. Sadequl Islam, Md. Kamruzzaman, Umme Kulsum Rima

**Affiliations:** ^1^ Department of Anatomy and Histology Faculty of Veterinary and Animal Science, Hajee Mohammad Danesh Science and Technology University Dinajpur Bangladesh; ^2^ Department of Dairy and Poultry Science Faculty of Veterinary and Animal Science, Hajee Mohammad Danesh Science and Technology University Dinajpur Bangladesh; ^3^ Department of Medicine Surgery and Obstetrics Faculty of Veterinary and Animal Science, Hajee Mohammad Danesh Science and Technology University Dinajpur Bangladesh

**Keywords:** adverse effects, mice, microplastics, thyroid function

## Abstract

**Background:**

Polystyrene microplastics (PS‐MPs) are pervasive pollutants impacting animals across ecosystems, including livestock and wildlife, through contaminated food, water, and air. MPs may disrupt endocrine function, particularly affecting the thyroid gland, which is essential for metabolism and development.

**Objectives:**

This study investigates the effects of PS‐MPs on thyroid function in mice, offering insights relevant to veterinary care by examining changes in gene expression and biochemical markers.

**Methods:**

PS‐MPs of 5 µm diameter were prepared in distilled water after probe sonication. Sixty male Swiss albino mice were divided into three groups: a control group and two treatment groups receiving 0.1 mg and 0.2 mg PS‐MPs via oral gavage for 28 days. Mice were anesthetised, and thyroid tissues were collected for histopathological, biochemical, and gene expression analyses. Biochemical tests included catalase, superoxide dismutase, reactive oxygen species, and hormone levels. Histopathology and gene expression (*TSHR* and *TPO*) of thyroid‐related genes were examined to assess PS‐MPs induced effects.

**Results:**

Exposure to PS‐MPs in mice led to significant increases in calcium, thyroxin, free T3, free T4, ALP, AST, ALT, and amylase levels, alongside elevated oxidative stress markers. Conversely, the levels of TSH, calcitonin, magnesium and phosphate decreased. Histopathological analysis showed abnormal thyroid follicle development, decrease parafollicular cells, with colloid loss, haemorrhage, and necrosis. Gene expression analysis revealed a marked reduction in *TSHR* and *TPO* levels in PS‐MPs treated groups, indicating thyroid dysfunction. These findings highlight the profound impact of PS‐MPs on thyroid gland function in mice.

**Conclusion:**

These findings underscore the potential risks that PS‐MPs pose to thyroid health, with potential consequences for other veterinary species. As environmental contamination rises, veterinarians may encounter more endocrine disorders linked to PS‐MPs, emphasising the need for further research and preventive measures.

## Graphical abstract

Exposure to PS‐MPs in mice resulted in significant alterations in thyroid function, marked by increased calcium, thyroxin, free T3, free T4, ALP, AST, ALT, and amylase levels, alongside elevated oxidative stress. Histopathological analysis revealed thyroid follicle abnormalities, including colloid loss, hemorrhage, and necrosis. Additionally, gene expression analysis showed reduced *TSHR* and *TPO* levels, indicating thyroid dysfunction, underscoring the profound impact of MPs on thyroid gland health in mice.

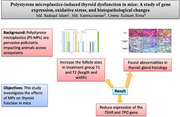

## 1 Introduction

Plastic pollution has become a significant environmental concern due to its widespread presence in terrestrial and marine ecosystems. Microplastics (MPs), defined as plastic particles smaller than 5 mm, are particularly alarming due to their presence in soil, water bodies, and air. These particles pose serious threats to various organisms, including aquatic species, birds, and mammals (Gallo et al. 2018; Laskar et al. 2019). Research suggests that MPs can interfere with the endocrine system, particularly affecting the thyroid gland, which is essential for metabolism, growth, and development (Amereh et al. 2019; Zhang et al. 2024). Environmental pollutants like MPs may impair thyroid function, leading to adverse health outcomes.

Studies have shown that MPs disrupt endocrine function across species. Zhao et al. (2020) reported that polystyrene microplastics (PS‐MPs) led to thyroid dysfunction in zebrafish, causing developmental defects and impaired growth. In mammals, MPs have been linked to hormone level disruptions, metabolic disorders, and reproductive issues (Kinlein et al. 2015). Laskar et al. (2019) emphasised that MPs can accumulate in organisms, disrupting hormonal regulation. MPs’ small size allows them to cross biological barriers, accumulate in tissues, and interfere with cellular functions (Kurunthachalam and Vimalkumar 2021). Despite these findings, studies on the effects of MPs on thyroid gene expression and histopathology in mammals, particularly mice, remain limited.

The mechanisms by which MPs affect thyroid function are still being explored. Evidence links MPs to oxidative stress and thyroid hormone disturbances in rats, impacting metabolism and reproductive health (Ullah et al. 2023). MPs have also been associated with altered thyroid‐stimulating hormone (TSH) levels, leading to thyroid dysfunction (Bereketoglu and Pradhan 2022). Research indicates that MPs can interfere with the hypothalamic‐pituitary‐thyroid (HPT) axis, which regulates thyroid homeostasis (Wright and Kelly 2017; Lopez et al. 2023). Prolonged exposure may result in thyroid dysfunction by affecting hormone synthesis and overall endocrine health.

PS‐MPs affect a wide range of species, including companion animals, livestock, and wildlife. Animals in agricultural, domestic, and wild environments are exposed to MPs through contaminated food, water, and air. Studying MPs effects on the endocrine system in model organisms, such as mice, can provide insights relevant to veterinary medicine. Endocrine disruption in animals can lead to metabolic disorders, developmental abnormalities, and reproductive issues. Investigating the gene expression, biochemical changes, and histopathological effects of MP‐induced thyroid dysfunction in mice can offer critical data for managing MP exposure in domestic and wild animal populations. As MP contamination increases, veterinarians may encounter more endocrine dysfunction cases, and findings from this study can help guide preventive and therapeutic measures. Most existing research has focused on hormonal alterations caused by MPs, with limited attention to the underlying molecular mechanisms, particularly thyroid gene expression and histopathology. Addressing this gap is critical for understanding the health risks associated with MP pollution. While some studies have explored the endocrine disruptions caused by MPs, little is known about how these particles affect thyroid function at the gene expression level and through histological changes. To fill this gap, this study aimed to provide a comprehensive understanding of MP‐induced thyroid dysfunction by evaluating gene expression, biochemical parameters, and histological characteristics in mice. In addition to thyroid‐specific measurements, systemic markers including alkaline phosphatase (ALP), aspartate aminotransferase (AST), alanine aminotransferase (ALT), amylase, and malondialdehyde (MDA)—were included to assess broader metabolic and liver function. Previous studies have demonstrated that MPs can lead to hepatic dysfunction and oxidative stress (Boran et al. 2024; Lee et al. 2024), which may further exacerbate thyroid disruption. Additionally, the potential for mineral imbalances, including altered levels of calcium, magnesium, and phosphate, was examined to explore the impact of MPs on mineral homeostasis, an essential factor in regulating thyroid function (Kannan and Vimalkumar 2021). Understanding these systemic effects is crucial for comprehensively assessing the risks posed by MP exposure on thyroid health and overall metabolic balance.

## 2 Materials And Methods

### 2.1 Microplastics

PS‐MPs with a particle diameter of 4.8–5 µm were sourced from Cospheric LLC, USA. Prior to experimentation, the PS‐MPs underwent a 30 min probe sonication process using an ultrasonic cleaner (Model: TUC‐32, China) to ensure uniform dispersion. Subsequently, the PS‐MPs were prepared in distilled water to achieve the desired concentration (pH 6.9 ± 0.1).

### 2.2 Experimental Animals

Six‐week‐old, healthy, male Swiss Albino mice (wild‐type, non‐genetically modified) were obtained from the Entomology Department at Rajshahi University. These mice were housed in a controlled environment maintained at 25°C temperature, with humidity levels ranging between 55–60% and a 12 h light/dark cycle. They were accommodated in stainless‐steel cages and allowed a two‐week acclimatisation period before commencement of the study. Throughout the experiment, the mice had ad libitum access to standard rodent feed and water. On commencement of the study, all mice weighed 25–30 g. All experimental protocols followed the guidelines set forth in the National Institutes of Health (NIH) guide for the care and use of laboratory animals. The protocol received approval from the Committee on the Ethics of the Institute of Research and Training, Hajee Mohammad Danesh Science and Technology University, under reference number BS/2024/11.

### 2.3 Doses and Administration

The mice were randomly divided into three groups of 20 mice each: one control group and two treatment groups exposed to 5 µm PS‐MPs. The exposure period lasted 28 days. The control group (Group C) received 0.5 mL of normal saline via oral gavage once daily.

Treatment Group T1 received 0.5 mL of an MP solution with a concentration of 0.1 mg/0.5 mL (equivalent to approximately 1.5 × 10⁶ particles) by oral gavage once daily for 28 days. This dosage was determined based on studies by Deng et al. (2017) and Haddadi et al. (2022). To assess potential dose‐dependent effects, Treatment Group T2 received 1.0 mL of the MP solution at 0.2 mg/mL, containing 0.2 mg of MPs (approximately 3.0 × 10⁶ particles), by oral gavage once daily.

### 2.4 Specimen Collection

On day 28 of the study, food was withheld for 6 h, and 1 mL of blood was then collected from 10 male BALB/c mice per treatment group for biochemical analysis. A 25‐gauge needle was used for blood collection. To minimise discomfort and in accordance with ethical protocols, the mice were then anaesthetised via intramuscular injection of ketamine at a dose of 35 mg/kg (Ketalar, Popular Pharmaceuticals Ltd., Bangladesh) combined with xylazine at a dose of 5–10 mg/kg (Xylaxin, Indian Immunologicals Ltd., India). After verifying deep anaesthesia, euthanasia was carried out via cervical dislocation following institutional guidelines. After euthanasia, blood samples were collected for biochemical studies, and thyroid gland tissues were precisely extracted for analysis. To preserve the structural integrity of the thyroid gland tissue, the samples were immediately immersed in modified Davidson's fluid (Biotech Concern, Bangladesh), comprising 30% formaldehyde solution, 15% ethanol, 5% glacial acetic acid, and 50% distilled water (Tian et al. 2024). Additionally, thyroid gland tissues were harvested for further examination, and a small portion (up to 10 mg) of the thyroid gland collected for RNA extraction was immediately preserved in an RNA protection reagent and stored at ‐80°C to ensure RNA integrity.

### 2.5 Biochemical Test

Thyroid tissues collected from the control group and PS‐MPs exposed groups were homogenised in phosphate‐buffered saline to obtain tissue lysates suitable for subsequent analyses. The biochemical analysis involved measuring catalase (CAT) activity using the supernatant from the thyroid homogenates, following the method described by Aebi (1984). A 50 µL aliquot of homogenate was mixed with 2 mL of phosphate buffered saline (pH 7.0), and then 1 mL of 30 mM H₂O₂ (in the same buffer) was added to initiate the reaction. The reaction mixture was immediately transferred to a quartz cuvette, and the decrease in absorbance was recorded at 240 nm using a UV‐visible spectrophotometer (Shimadzu UV‐1800, Japan) in kinetic mode for 1 min. The rate of decrease in absorbance (ΔA240/min) was used to calculate CAT activity, where a 0.01 unit decrease per minute corresponded to one unit (U) of enzyme activity.

Superoxide dismutase (SOD) activity was measured using the method described by Kakkar et al. (1984). The reaction mixture contained 1.2 mL of sodium pyrophosphate buffer (0.052 mM, pH 7.0), 0.1 mL of 186 mM phenazine methosulfate, and 0.3 mL of the supernatant from the centrifuged thyroid homogenate. The reaction was initiated by adding 0.2 mL of 780 mM NADH and stopped with 1 mL of glacial acetic acid. The chromogen formed was extracted, and its absorbance was measured at 560 nm using a spectrophotometer (Shimadzu UV‐1800, Japan). The enzyme activity was calculated from the inhibition of chromogen formation and expressed in units per milligram of protein.

Peroxidase (POD) activity was determined using the method of Chance and Maehly (1954). The assay mixture consisted of 0.1 mL guaiacol, 2.5 mL phosphate buffer (pH 5.0), 0.3 mL of 30 mM H₂O₂, and 0.1 mL of homogenate. The reaction mixture was incubated at room temperature, and the increase in absorbance at 470 nm due to the oxidation of guaiacol was monitored every 15 seconds for 1 min using a UV‐visible spectrophotometer in kinetic mode. One unit of POD activity was defined as the amount causing a 0.01 increase in absorbance per minute.

ROS levels were quantified following the protocol outlined by Hayashi et al. (2007). The assay involved mixing 5 mL of tissue homogenate with 140 mL of 0.1 M sodium acetate buffer (pH 4.8) and distributing the mixture into 96‐well plates. After incubating at 37°C for 5 min, 100 mL of ferrous sulphate and N, N‐diethyl‐para‐phenylenediamine solution was added to each well, followed by a further incubation at 37°C for 1 min. Absorbance was measured at 505 nm over 180 seconds at 15‐second intervals using a microplate reader. A standard curve was constructed from these data, and ROS levels were determined in units per milligram of tissue, with one unit of ROS corresponding to 1.0 milligram per litre of hydrogen peroxide (H₂O₂) in the sample. Whole blood was separated by centrifugation at 3,000 rpm for 10 min, and the serum collected.

The serum T4 (Thyroxine ELISA Kit, Elabscience Biotechnology Inc., USA), TSH, free T3, and free T4 (TSH‐ELISA Kit, Free T3‐ELISA Kit, Free T4‐ELISA Kit, MyBioSource Inc., USA) levels were quantified using highly sensitive ELISA kits, following the manufacturer's protocols. Briefly, 50 µL of serum was added to pre‐coated microtiter wells, incubated, washed, and treated with a biotinylated secondary antibody. After adding the substrate solution, the enzymatic reaction was measured at 450 nm. Hormone concentrations were calculated using standard curves generated from known concentrations.

Serum calcitonin levels were determined using a commercially available ELISA kit (Mouse Calcitonin ELISA Kit, Antibodies.com Limited, UK), specifically designed for murine calcitonin detection. A 50 µL serum sample was added to wells of a pre‐coated plate, followed by incubation and washing. A secondary enzyme‐conjugated antibody was added, and the reaction was developed using a substrate solution. Absorbance was measured at 450 nm, and calcitonin concentrations were calculated from the standard curve.

The enzymatic activities of ALP, AST, ALT, and amylase in the serum were measured using the Siemens Advia 1800 system, ensuring precise calibration and proper sample handling. The standard protocols for each enzyme were followed according to the instructions of the manufacturer.

To determine the level of MDA in mice blood, the method described by Nielsen et al. (1997), which utilises the thiobarbituric acid (TBA) assay, was followed. In this procedure, 100 µL of serum was combined with 900 µL of distilled water and 500 µL of TBA reagent. The mixture was heated at 100°C for one hour, then allowed to cool before being centrifuged at 4000 rpm for 10 min. The absorbance of the resulting supernatant was measured at 534 nm using a spectrophotometer, and the MDA concentration was determined and expressed in micromoles per litre.

For the assessment of calcium, magnesium, and phosphate homeostasis, serum levels of these electrolytes were measured using the Siemens Advia 1800 photometric analyser. The Siemens Advia 1800 was calibrated with standard solutions, and serum samples along with reagents were loaded according to the manufacturer's instructions. Photometric measurements were automatically recorded, with quality control samples ensuring the accuracy of the results.

### 2.6 Histopathology

Formalin was carefully removed from the tissues, which were then dehydrated using a graded alcohol series, starting at 70% and progressing through 80%, 90%, 95%, and 100% concentrations, with a minimum of one hour at each step, following the protocol outlined by Islam et al. (2023). The tissues were then treated with xylene, first in xylene‐1 for 90 min, followed by xylene‐2 for an additional 90 min. After xylene treatment, the tissues were immersed in liquid paraffin at 60°C for 90 min before cooling and embedding them into paraffin blocks. These blocks were sectioned into 6 µm slices using a LEICA RM2125 RTS microtome. The sections were floated on water at 45°C, mounted on clean, oil‐free glass slides, and dried in an oven at 62°C for 20 min. Haematoxylin and eosin (H&E) staining was performed, and the slides were sealed with Canada balsam, following the procedures described by Drury et al. (1983). High‐resolution images of the stained tissues were captured using an Amscope (MA500) camera attached to a Richter Ptica U‐2T microscope at 10×, 40×, and 100× magnifications. To analyse parafollicular cells in the thyroid follicles, ImageJ software was employed using the cell counting method described by Curvo et al. (2020) and the field length specified by Islam et al. (2023) for consistency. For histomorphometric analysis, one representative histological section per animal was analysed, with a total of 10 sections per group to ensure consistency in comparisons. A prior work (Islam et al. 2024) utilised a cell field of a certain length to count parafollicular cells. All measured follicle lengths and widths were analyzed quantitatively, and the data were presented following the approach described by Müller et al. (2011).

### 2.7 Evaluation of Gene Expression Due to MP Exposure

For RNA extraction, the stored thyroid tissues were pulverised and mixed with liquid nitrogen to ensure thorough homogenisation. RNA was then extracted using the Monarch Total RNA Miniprep Kit (Cat No. T2010S, New England Biolabs Inc.), with a slight modification to the standard protocol by incorporating liquid nitrogen. Each group underwent three independent extractions. After RNA extraction, complementary DNA (cDNA) was synthesised using the ProtoScript II First Strand cDNA Synthesis Kit (Cat No: E6560S, New England Biolabs Inc.). The purity and concentration of the RNA were assessed using the NANODROP ONE (Thermo Scientific, USA). For the RT‐qPCR, the Luna Universal qPCR Master Mix (Cat No: M3003S, New England Biolabs Inc.) and the qTOWER3G Real‐Time PCR system (Analytik Jena) were employed. The thermal cycling protocol included an initial denaturation at 95°C for 2 min, followed by cycles of denaturation at 95°C for 5 seconds, annealing at 62°C for 30 seconds, and extension at 72°C for 30 seconds.

Gene expression of TSHR and TPO was evaluated using RT‐qPCR, with HPRT1 serving as the housekeeping gene. HPRT1 was chosen as the reference gene because of its consistent expression across samples. The CT (cycle threshold) values of HPRT1 were used to normalise the expression of the target genes. The ΔCT for each sample was obtained by subtracting the CT value of HPRT1 from the CT value of the gene of interest (GOI), following the formula: ΔCT = CT_GOI_—CT *_HPRT1_*. This process normalised the expression of the GOI relative to HPRT1. The average ΔCT value of the control samples was then calculated as the baseline. The ΔΔCT for each experimental sample was derived by subtracting the average ΔCT of the control group from the sample's ΔCT value, using the formula: ΔΔCT = ΔCT_Sample_—ΔCT *_HPRT1_*_(average)._ Finally, the relative expression of the gene was determined using the formula 2^−ΔΔCT^, which indicates the fold change in the expression of the GOI in experimental samples compared to control samples (Livak and Schmittgen 2001).

The primer sequences for the targeted genes were as follows: *TSHR* (forward: ACA AAG CTG GAT GCT GTT TACC; reverse: GGG CAT AAG GAC GGC AGA AT; product size: 171 bp) (Gerhardt et al. 2023), *TPO* (forward: AGC TCA AGA CAC TGG ACA GGAAC; reverse: CAA TGT CTG GCT CCA AAG CAG; product size: 88 bp), and the housekeeping gene *HPRT1* (forward: GTT GGG CTT ACC TCA CTG CT; reverse: TCA TCG CTA ATC ACG CT; product size: 132 bp) (Gerhardt et al. 2023).

### 2.8 Statistical Analysis

Data analysis was conducted using SPSS version 16 (SPSS, Inc.). Initially, a normality test (Shapiro–Wilk test) was performed to evaluate the distribution of the data. To assess differences between group means, one‐way ANOVA was employed, with statistical significance defined as *p* < 0.05. If ANOVA revealed significant differences, Tukey's Honestly Significant Difference (HSD) post‐hoc test was used to pinpoint the specific group comparisons that contributed to the observed differences.

## 3 Results

### 3.1 Changes in Biochemical Markers

The biochemistry results and comparisons between the study groups are shown in Table 1. In summary, significant differences (p < 0.001) were found between all three study groups for all tested parameters. Thyroxin, ALP, AST, ALT, amylase, malondialdehyde and calcium were higher in both the T1 and T2 groups compared to the control group and were also higher in the T2 group compared with T1. Conversely, CAT, SOD magnesium, phosphate and calcitonin were lower in both the T1 and T2 groups compared to the control group and were also higher in the T2 group compared with T1.

**TABLE 1:** Impact of various concentrations of PS‐MPs (Control, 0.1 mg/0.5 mL, and 0.2 mg/1 mL) on thyroid function, enzyme activity and oxidative stress in mice.

Parameters	Control (C)	T1 (0.1 mg/0.5 mL)	T2 (0.2 mg/1 mL)	*p*‐Value	*f*‐ratio value
Thyroxin (µg/dL)	4.21 ± 1.0723^a^	12.48 ± 2.1989^b^	15.35 ± 2.1288^c^	< 0.001	*F* (2, 27) = 86.44
TSH(ng/mL)	0.717 ± 0.1398^a^	0.138 ± 0.0961^b^	0.046 ± 0.032^c^	< 0.001	*F* (2, 27) = 133.11
Free T3(ng/mL)	0.622 ± 0.2243^a^	1.994 ± 0.1491^b^	2.276 ± 0.1922^c^	< 0.001	*F* (2, 27) = 214.51
Free T4(ng/mL)	1.142 ± 0.3667^a^	2.412 ± 0.1136^b^	2.612 ± 0.1136^c^	< 0.001	*F* (2, 27) = 118.99
Calcitonin (pg/mL)	13.59 ± 0.9803^a^	6.674 ± 0.5105^b^	2.298 ± 0.8182^c^	< 0.001	*F*(2, 27) = 514.21
ALP (U/L)	84.96 ± 4.1559^a^	114.1 ± 5.5644^b^	125.36 ± 4.4699^c^	< 0.001	*F* (2, 27) = 191.16
AST (U/L)	51.07 ± 4.0395^a^	65.95 ± 2.9744^b^	72.95 ± 3.3304^c^	< 0.001	*F* (2, 27) = 103.31
ALT (U/L)	34.99 ± 2.9403^a^	44.14 ± 1.9225^b^	50.35 ± 2.4994^c^	< 0.001	*F*(2, 27) = 96.35
Amylase (U/L)	716.55 ± 21.9252^a^	760.16 ± 9.1101^b^	772.43 ± 7.3455^c^	< 0.001	*F* (2, 27) = 41.89
Malondialdehyde (n mol/L)	1.481 ± 0.4577^a^	5.628 ± 0.7888^b^	7.371 ± 0.5908^c^	< 0.001	*F* (2, 27) = 232.60
CAT (U/mg protein)	10.22 ± 1.8576^a^	7.01 ± 1.2991^b^	4.46 ± 0.8127^c^	< 0.001	*F* (2, 27) = 43.09
SOD (U/mg protein)	54.97 ± 4.1344^a^	29.41 ± 3.1817^b^	24.074 ± 1.6996^c^	< 0.001	*F* (2, 27) = 271.77
POD (nmole)	12.39 ± 1.8894^a^	8.324 ± 0.848^b^	6.27 ± 0.9627^c^	< 0.001	*F* (2, 27) = 55.79
ROS (U/mg tissue)	0.544 ± 0.0772^a^	1.443 ± 0.2313^b^	2.002 ± 0.4449^c^	< 0.001	*F* (2, 27) = 63.06
Calcium (mg/dL)	9.4 ± 0.5375^a^	10.47 ± 0.6165^b^	11.95 ± 0.3028c	< 0.001	*F* (2, 27) = 64.66
Magnesium (mg/dL)	1.94 ± 0.1897^a^	1.6 ± 0.1491^b^	1.3 ± 0.1491^c^	< 0.001	*F*(2, 27) = 38.23
Phosphate (mg/dL)	3.43 ± 0.6056^a^	2.24 ± 0.1174^b^	1.94 ± 0.1174^c^	< 0.001	*F*(2, 27) = 47.24

*Note*: Values are shown as Mean ± SD (*n* = 10/group). Means within the same row for each parameter that carry different superscripts (^a,b,c^) differ significantly at *p* < 0.05, as determined by one‐way ANOVA, followed by Tukey's HSD post hoc test.

### 3.2 Impact of Various Concentrations of PS‐MPs on Thyroid Gland Development in Mice

**FIGURE 1:**
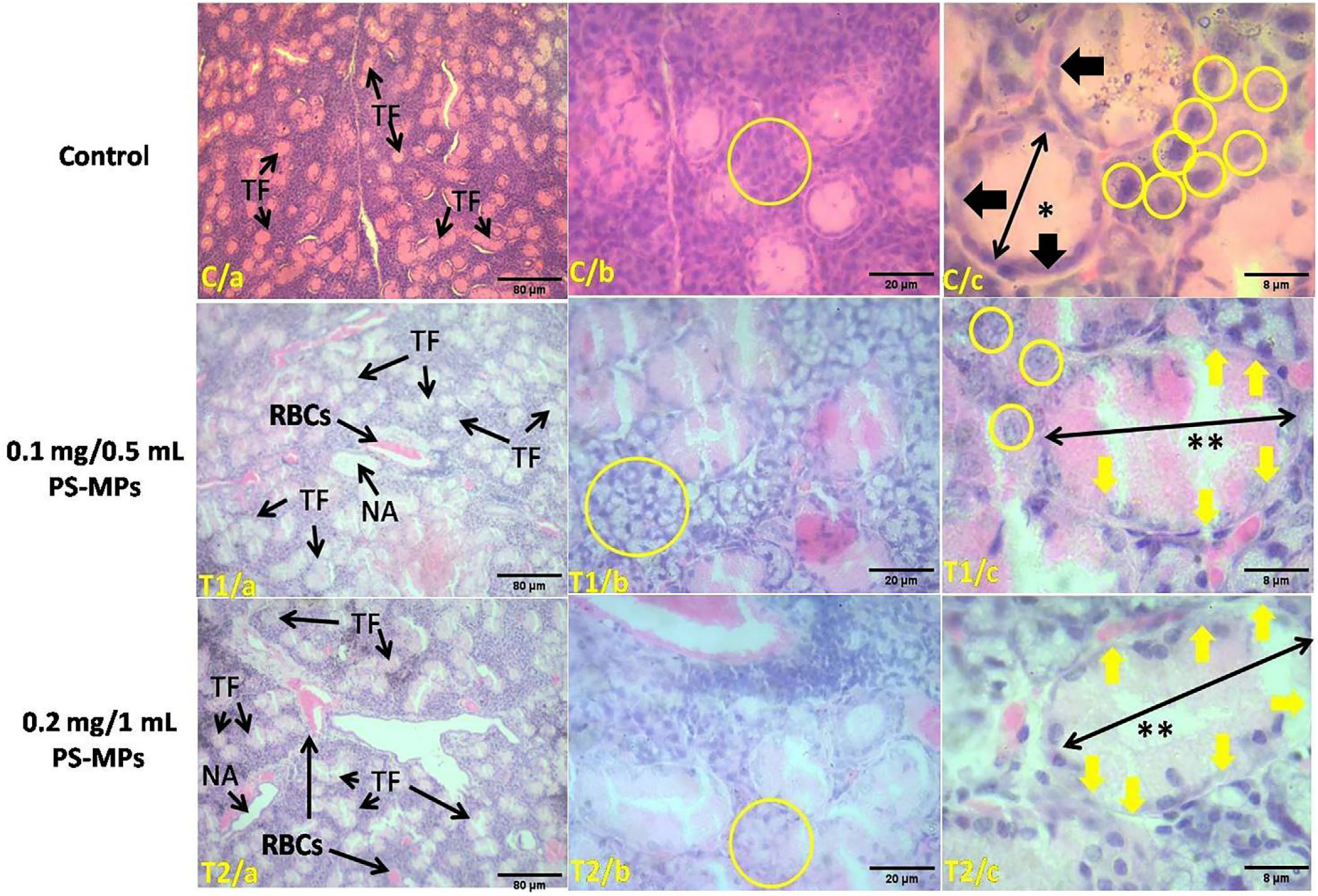
Impact of different concentrations of PS‐MPs on the thyroid gland in mice. The figure presents histological analysis at various magnifications (10×, 40×, and 100×) with H&E staining and scale bars of 80 µm, 20 µm, and 8 µm, respectively. Control Group (C/a, C/b, C/c): Panels show normal thyroid morphology with well‐formed thyroid follicles (TH) filled with colloid (*). The follicular epithelium displays intact cellular junctions (black arrows in C/c), and parafollicular cells are densely populated (yellow circles). T1 Group (T1/a, T1/b, T1/c; 0.1 mg/0.5 mL PS‐MPs): Displays show altered thyroid follicles (TH), including haemorrhage, presence of RBCs, necrotic areas (NA), disrupted cellular junctions (yellow arrows in T1/c), and decreased parafollicular cell density (yellow circles). Colloid depletion is visible (**). T2 Group (T2/a, T2/b, T2/c; 0.2 mg/1 mL PS‐MPs): Similar abnormalities to T1, including more extensive colloid loss (**), disrupted follicular structure, and reduced parafollicular cell presence (yellow circles).

We observed the impact of different PS‐MPs concentrations (control, 0.1 mg/0.5 mL, and 0.2 mg/1 mL) on thyroid gland development in mice under various magnifications (10×, 40×, and 100×) using H&E stain. In control samples (C/a, C/b, and C/c), normal thyroid morphology is observed, with with well‐formed thyroid follicles (TF) filled with colloid (*) (Figure 1). The follicular epithelium shows continuous cellular junction alignment (black arrow in C/c), and there is a dense presence of parafollicular cells (yellow circle). In samples exposed to 0.1 mg/0.5 mL PS‐MPs (T1/a, T1/b, and T1/c), abnormalities such as hemorrhage, presence of RBCs, and necrotic areas (NA) are evident. The altered thyroid follicles (TF) exhibit partial colloid loss (**), disrupted cellular junctions (yellow arrow in T1/c), and reduced parafollicular cell density (yellow circle). Similarly, in samples exposed to 0.2 mg/1 mL PS‐MPs (T2/a, T2/b, and T2/c), there is evidence of hemorrhage, presence of Red Blood Cells (RBCs), and necrotic areas (NA), along with partial depletion of colloid (**), disrupted cellular junction alignment (yellow arrow in T2/c), and decreased parafollicular cell density (yellow circle) (Figure 1). The study observed a significant reduction in the parafollicular cell count of the thyroid gland in mice exposed to different concentrations of PS‐MPs, providing evidence for the decrease in parafollicular cell density. The control group (C) had the highest cell count (47.4 ± 1.43), followed by the T1 group (0.1 mg/0.5 mL) with a count of 25.7 ± 0.88, and the T2 group (0.2 mg/1 mL) showing the lowest count (18.9 ± 0.95) (Table 2). The differences in cell counts between the groups were statistically significant (*p* < 0.001).

**TABLE 2:** Impact of various concentrations of PS‐MPs (Control, 0.1 mg/0.5 mL, and 0.2 mg/1 mL) on parafollicular cell count of thyroid gland in mice.

Parameters	Control (C)	T1 (0.1 mg/0.5 mL)	T2 (0.2 mg/1 mL)	*p*‐value	*f*‐ratio value
Parafollicular cell	47.4 ± 1.4298^a^	25.7 ± 0.8756^b^	18.9 ±0.9487^c^	<0.001	*F*(2, 27) = 1791.08,

*Note*: Values are shown as Mean±SD (*n* = 10/group). Means within the same row for each parameter that carry different superscripts (^a,b,c^) differ significantly at *p* < 0.05, as determined by one‐way ANOVA, followed by Tukey's HSD post hoc test.

Table 3 summarizes the mean follicle length and width across control and PS‐MP‐treated groups. A significant, dose‐dependent increase in both follicle length and width was observed in the treated groups compared to control (*p* < 0.001). The highest values were recorded in the T2 group (290.08 ± 58.98 µm length; 218.14 ± 19.97 µm width), indicating pronounced follicular hypertrophy at the higher PS‐MP concentration. Statistical analysis confirmed that all group means differed significantly, as indicated by distinct superscripts and supported by one‐way ANOVA followed by Tukey’s HSD post hoc test.

**TABLE 3:** Summary of thyroid follicle dimensions across treatment groups due to the impact of various concentrations of PS‐MPs (Control, 0.1 mg/0.5 mL, and 0.2 mg/1 mL) in mice.

Group	Mean length (µm) ± SD	Mean width (µm) ± SD
Control (C)	193.21 ± 29.39^a^	149.66 ± 32.88^a^
T1 (0.1 mg/0.5 mL)	270.18 ± 49.48^b^	208.58 ± 27.26^b^
T2 (0.2 mg/1 mL)	290.08 ± 58.98^c^	218.14 ± 19.97^c^
*p*‐Value	< 0.001	< 0.001
*f*‐ratio value	*F*(2, 27) = 34.70	*F(*2, 27) = 55.686

*Note*: Values are shown as Mean±SD (n = 30/group). Means within the same column for each parameter that carry different superscripts (^a,b,c^) differ significantly at *p* < 0.05, as determined by one‐way ANOVA followed by Tukey's HSD post hoc test.

**FIGURE 2:**
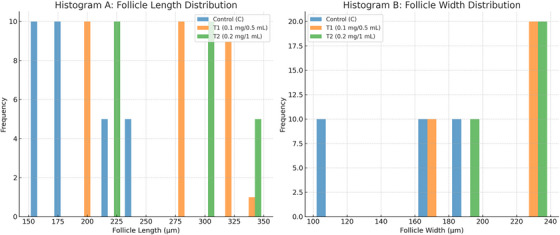
Histograms of thyroid follicle dimensions across groups. **(A) Follicle length distribution**: Histogram showing the distribution of follicle lengths (µm) in the thyroid glands of mice from the Control group, T1 (0.1 mg/0.5 mL PS‐MPs), and T2 (0.2 mg/1 mL PS‐MPs). A clear shift toward larger follicle lengths is observed in the treated groups, particularly in T2, indicating follicular hypertrophy due to PS‐MP exposure. **(B) Follicle width distribution**: Histogram illustrating the distribution of follicle widths (µm) across the same experimental groups. Similar to follicle length, an increase in follicle width is evident in the treated groups compared to control, further supporting morphological changes associated with PS‐MPs exposure.

Figure 2 presents histograms illustrating the distribution of thyroid follicle dimensions across experimental groups. Both follicle length (A) and width (B) show a noticeable shift toward larger values in the PS‐MP‐treated groups (T1 and T2). The histograms visually reinforce the statistically significant differences (*p* < 0.001) in follicle size across all groups, as detailed in Table 3, highlighting the significant impact of PS‐MPs on thyroid follicle development.

### 3.3 TSHR and TPO Gene Expression

**FIGURE 3:**
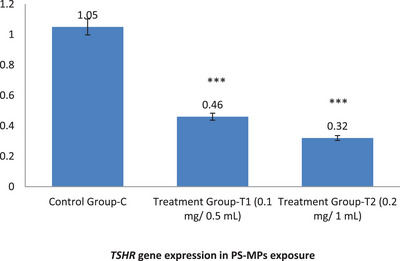
This figure illustrates the impact of PS‐MPs exposure on the expression of the *TSHR* gene in mice. Groups T1 and T2, exposed to MPs, exhibit a significant reduction in *TSHR* gene expression levels compared to the untreated control group (C). Statistical analysis (*p* < 0.05) reveals significant differences (***) between the treatment groups and the control group, as well as between the treatment groups themselves. These findings highlight the detrimental effects of MP exposure on *TSHR* gene expression.

**FIGURE 4:**
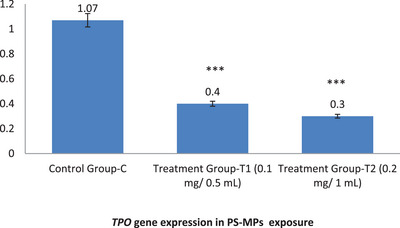
This figure illustrates the impact of PS‐MPs exposure on the expression of the *TPO* gene in mice. Groups T1 and T2, exposed to PS‐MPs, show a marked decrease in *TPO* gene expression levels compared to the untreated control group (C). Statistical analysis (*p* < 0.05) confirms significant differences (***) between the treatment groups and the control group, as well as between the treatment groups themselves. This underscores the harmful effects of PS‐MPs exposure on *TPO* gene expression.

In the control group (C), the expression levels of *TSHR* and *TPO* were 1.05 and 1.07, respectively. However, in the treatment group exposed to 0.1 mg/0.5 mL PS‐MPs (T1), the expression levels of *TSHR* and *TPO* significantly decreased to 0.46 and 0.4, respectively. In the treatment group exposed to 0.2 mg/1 mL PS‐MPs (T2), the expression levels further dropped to 0.32 for *TSHR* and 0.3 for *TPO*. These results indicate a substantial reduction in the expression of both genes due to PS‐MPs exposure, underscoring the adverse effects of PS‐MPs on gene regulation in mice (Figure 3 and Figure 4).

## 4 Discussion

The increasing exposure of animals to environmental pollutants, particularly PS‐MPs, raises significant concerns regarding their health and well‐being. As companion animals, livestock, and wildlife face growing risks from PS‐MPs induced endocrine disruption, this research provides valuable insights that could inform preventive strategies and therapeutic approaches in veterinary practice. Understanding the biochemical, genetic, and morphological impacts of PS‐MPs on the thyroid is essential for addressing the broader health risks posed to animals in both domestic and ecological settings.

The selection of PS‐MPs with a particle size of approximately 5 µm in this study was based on increasing evidence that microplastics of this size range are widely present in various environmental matrices, including aquatic systems, soil, and even air. Studies have reported the presence of small‐sized microplastics (1–10 µm) in drinking water, seafood, and atmospheric fallout, which are potential exposure routes for both wildlife and humans (Cox et al. 2019; Zhang et al. 2020). Importantly, particles smaller than 10 µm have a higher propensity to cross biological barriers, such as the gastrointestinal lining or even the blood–brain barrier, leading to potential systemic distribution and organ‐specific accumulation (Yong et al. 2020). Furthermore, the concentration of PS‐MPs used in this study was selected to mimic environmentally relevant exposure levels while maintaining experimental detectability and reproducibility, as suggested in previous toxicological studies (Lu et al. 2016; Deng et al. 2017).

The biochemical changes observed in this study indicate significant disruptions in thyroid function and general metabolic health in mice. Elevated levels of thyroxine (T4) in both treatment groups suggest that PS‐MPs exposure may trigger hyperthyroidism. This is likely due to the endocrine‐disrupting properties of PS‐MPs, which interfere with thyroid hormone synthesis and release pathways. These findings are consistent with previous studies demonstrating that MPs can act as hormone mimics or disruptors, affecting thyroid regulation (Kannan and Vimalkumar 2021).

Additional experiments measuring TSH levels in PS‐MPs exposed animals revealed a significant decrease in TSH compared to controls, supporting the notion of hyperthyroidism. Low TSH levels are typically associated with hyperthyroidism, as TSH is a key regulator of thyroid hormone production (Baliram et al. 2012). While reduced TSH levels in the PS‐MPs exposed groups suggest suppressed feedback, the increased levels of thyroid hormones (free T3 and free T4) in these animals further support the conclusion of hyperthyroidism. This altered TSH response may be indicative of dysfunction within the HPT axis, which warrants further investigation to better understand the underlying mechanisms.

The observed increase in T4 levels, despite the decreased expression of *TPO* and *TSHR*, presents an intriguing paradox, as both *TPO* and *TSHR* play vital roles in thyroid hormone synthesis and secretion. This discrepancy could be due to a compensatory response to the altered hormonal environment caused by PS‐MPs exposure. The downregulation of *TSHR* may indicate a reduced sensitivity or response to *TSH* signalling, which typically stimulates T4 production. However, the increased T4 levels may reflect disruptions in thyroid regulation, potentially due to impaired negative feedback mechanisms or alterations in other signalling pathways that are not directly dependent on *TSHR*. Studies (Baliram et al. 2012)​ have shown that low *TSH* levels and the loss of *TSHR* signalling can disrupt normal thyroid hormone regulation and bone homeostasis, suggesting that similar mechanisms could be at play in this context. These findings suggest that PS‐MPs exposure could be influencing thyroid function in a complex manner, involving factors beyond the traditional TSH‐TSHR‐TPO pathway. Further investigation is required to elucidate the intricate interactions between these elements in PS‐MPs exposed animals.

The observed reduction in parafollicular cell density in PS‐MPs exposed animals suggests impaired calcitonin production. While calcitonin is involved in calcium homeostasis, its role is secondary compared to other mechanisms, particularly those regulating calcium through the parathyroid gland and kidney. The decrease in parafollicular C cell density may reflect disruptions in thyroid function that could contribute to disturbances in calcium regulation (Davey and Findlay 2013). Changes in calcium and phosphate levels could result from multiple factors, including direct toxicity, inflammation, and potential impacts on renal function. Further studies are needed to clarify how PS‐MPs exposure affects calcium balance through these interconnected systems.

The morphological changes observed in the thyroid gland in response to PS‐MPs exposure further support the conclusion that PS‐MPs induce oxidative stress, leading to cellular damage and altered thyroid function. These changes, including haemorrhage, necrotic areas, and colloid depletion, are indicative of thyroid dysfunction. These findings align with previous studies showing that PS‐MPs can interfere with endocrine function by generating reactive oxygen species (ROS) and disrupting cellular homeostasis (Ullah et al. 2023; Hua et al. 2022).

The reduction of parafollicular C cells in mice exposed to PS‐MPs can be attributed to oxidative stress, inflammation, and direct cellular toxicity. C cells are particularly vulnerable to PS‐MPs induced damage. The decrease in calcitonin secretion could explain the significant increase in calcium levels observed in the PS‐MPs exposed animals, as the body's ability to regulate calcium is compromised. Additionally, the partial colloid loss observed in the thyroids of treated animals suggests that PS‐MPs disrupt follicular integrity, affecting hormone storage. Similar disruptions have been reported in aquatic species exposed to PS‐MPs, reinforcing the connection between PS‐MPs and endocrine disruption (Al‐Thawadi 2020; Witczak et al. 2024).

The dose‐dependent increase in thyroid follicle size observed with higher concentrations of PS‐MPs further supports the hypothesis that PS‐MPs disrupt thyroid function. The enlargement of thyroid follicles may result from oxidative stress or inflammation induced by PS‐MPs, which leads to follicular hyperplasia. These findings are consistent with previous studies that highlighted PS‐MPs as endocrine disruptors capable of inducing significant morphological changes in hormone‐related organs (Poncin et al. 2008; Kochman et al. 2021; Mittal et al. 2023). Similar thyroid alterations in fish exposed to PS‐MPs were also reported in studies by Guerrera et al. (2021) and Ullah et al. (2023). Our study extends these findings by demonstrating a dose‐dependent effect of MPs in mammals, emphasising the threat PS‐MPs pose to thyroid health.

The downregulation of TSHR and TPO gene expression in PS‐MPs exposed mice indicates a detrimental impact on thyroid function. The reduced expression of TSHR could impair TSH signalling, leading to decreased thyroid hormone production, while the decreased expression of TPO suggests a further inhibition of thyroid hormone synthesis (Marin‐Sanchez et al. 2019; Kim et al. 2021). These findings are consistent with previous studies showing that environmental pollutants like PS‐MPs disrupt thyroid homeostasis by interfering with hormone synthesis and gene regulation (Ullah et al. 2023; Hua et al., 2022). Other studies have demonstrated that exposure to endocrine‐disrupting chemicals, such as bisphenol A, leads to significant downregulation of thyroid‐related genes, suggesting that MPs may share similar mechanisms of action (Vom Saal and Vandenberg 2021; Liu et al. 2024).

## 5 Conclusion

In conclusion, the results highlight the significant adverse effects of PS‐MPs exposure on thyroid gland function in mice, with important implications for other veterinary species. We observed marked increases in thyroid hormone thyroxin (T4) levels and liver enzymes (ALP, AST, ALT, amylase), indicating disrupted thyroid function and potential liver stress. Structural changes in thyroid follicles, such as haemorrhage and reduced parafollicular cell density, further support the physiological disturbances caused by PS‐MPs. These findings align with previous research across various species, underscoring the broader environmental health threat posed by PS‐MPs. The downregulation of *TSHR* and *TPO* gene expression emphasises the interference of PS‐MPs with thyroid hormone synthesis and signalling pathways. Future research should focus on elucidating the precise mechanisms behind these disruptions and evaluating the long‐term impacts of PS‐MPs exposure on thyroid health, with relevance not only to human health but also to the well‐being of animals exposed to these pollutants in natural and agricultural ecosystems.

## Author Contributions

**Md. Sadequl Islam**: conceptualisation, data curation, methodology, investigation, supervision, writing ‐ original draft, writing ‐ review and editing. **Md. Kamruzzaman**: conceptualisation, investigation, writing ‐ original draft, writing ‐ review and editing. **Umme Kulsum Rima**: conceptualisation, methodology, writing ‐ review and editing.

## Ethical Statement

The protocol received approval from the Committee on the Ethics of the Institute of research and Training, Hajee Mohammad Danesh Science and Technology University, under reference number BS/2024/11.

## Conflicts of Interest

The authors declare no conflicts of interest.

### Peer Review

The peer review history for this article is available at https://www.webofscience.com/api/gateway/wos/peer‐review/10.1002/vms3.70393.

## Data Availability

Data will be provided upon request from the corresponding author.
